# Comparison of Transcranial Direct Current Stimulation Electrode Montages for the Lower Limb Motor Cortex

**DOI:** 10.3390/brainsci9080189

**Published:** 2019-08-06

**Authors:** Radhika Patel, Sangeetha Madhavan

**Affiliations:** Department of Physical Therapy, University of Illinois at Chicago (UIC), Chicago, IL 60612, USA

**Keywords:** tDCS electrodes, corticomotor excitability, lower limb

## Abstract

Transcranial direct current stimulation (tDCS) has been widely explored as a neuromodulatory adjunct to modulate corticomotor excitability and improve motor behavior. However, issues with the effectiveness of tDCS have led to the exploration of empirical and experimental alternate electrode placements to enhance neuromodulatory effects. Here, we conducted a preliminary study to compare a novel electrode montage (which involved placing 13 cm^2^ electrodes anterior and posterior to the target location) to the traditionally used electrode montage (13 cm^2^ stimulating electrode over the target area and the 35 cm^2^ reference electrode over the contralateral orbit). We examined the effects of tDCS of the lower limb motor area (M1) by measuring the corticomotor excitability (CME) of the tibialis anterior muscle using transcranial magnetic stimulation in twenty healthy participants. We examined behavioral effects using a skilled motor control task performed with the ankle. We did not find one electrode montage to be superior to the other for changes in the CME or motor control. When the group was dichotomized into responders and non-responders (based on upregulation in CME), we found that the responders showed significant upregulation from baseline after tDCS for both montages. However, only the responders in the traditional montage group showed significant changes in motor control after tDCS. These results do not support the superiority of the new anterior–posterior montage over the traditional montage. Further work with a larger cohort and multiple cumulative sessions may be necessary to confirm our results.

## 1. Introduction

Transcranial direct current stimulation (tDCS), a promising non-invasive neuromodulatory technique, has been studied for over two decades in individuals with and without neurological disorders [1,2,3]. Numerous studies have reported moderate effects with tDCS to augment practice-related cognitive and motor behavior [3,4,5], yet others have reported contrasting results, which includes high inter-individual variability and low intra-subject reliability [6,7,8,9,10]. Factors that influence the effects of tDCS can be broadly classified into modifiable elements, which include current intensity, size of electrodes, electrode placement, duration of stimulation, and non-modifiable elements, such as cerebral anatomy, psychology, genetics, age, severity of existing neurological impairments, and other such biological factors. The growth of conflicting evidence concerning the reliability and extent of neuromodulation of tDCS effects has resulted in numerous studies exploring alternative parameters to optimize the effects of stimulation [11,12,13,14,15].

The conventional electrode montage for the upper and lower limb primary motor cortex (M1) consists of the center of the stimulating electrode placed over the target region and the reference or inactive electrode over the contralateral supraorbital region [16]. A few empirical studies have suggested that the traditional electrode configuration may be suboptimal to stimulate the M1 as the field strength is not directly below the target electrode and has been hypothesized to be between the target and reference electrodes [17,18]. Based on the results of finite element modelling with numerous electrode configurations, Rampersad et al. (2014) proposed an alternate electrode montage for the M1, with the anode 5 cm posterior and the cathode 5 cm anterior to the motor cortex. This configuration predicted an 88% higher mean electric field strength than that achieved with standard configurations [18]. However, this proposed electrode montage has not been tested experimentally in humans. In the present study, we aimed to test the hypothesis that an alternate electrode montage, which involves placing the anode and reference electrode peripheral to the target area, is superior to the conventional M1 montage. The conventional electrode placement for the lower limb M1 involves placing the active electrode (13 cm^2^) on the motor hotspot and the reference electrode (35 cm^2^) over the contralateral supra-orbital region. Rampersad et al. (2014) used two large 35 cm^2^ electrodes in their study, which modelled current distributions for the upper limb M1, but we intentionally chose to use a smaller active electrode typically used in lower limb tDCS studies [19,20,21,22]. We examined the neuromodulatory effects of 1 mA of tDCS for 15 min on the lower limb M1 by measuring the corticomotor excitability of the tibialis anterior muscle using transcranial magnetic stimulation (TMS), and we further examined behavioral effects using a skilled motor control task performed with the ankle.

## 2. Materials and Methods

### 2.1. Participants

A convenience sample of twenty healthy volunteers (12 males, 8 females; 24.15 ± 3 years) participated in the study. A verbal description of the study and the methods involved was provided, and a written informed consent approved by the Institutional Review Board of the University of Illinois at Chicago was obtained. Prior to inclusion, all participants were screened for contraindications to TMS, which included the presence of metal implants, a history of seizures, medications that alter the central nervous system’s excitability, recent trauma/fracture/surgery of the skull, and pregnancy [23]. Leg dominance for all participants was determined by asking them with which leg they would prefer to kick a ball. Participants were instructed to refrain from alcohol/caffeine intake for 24 h before the scheduled TMS/tDCS session [24].

### 2.2. Experimental Study Design

We chose a crossover design, where each participant attended two block-randomized tDCS sessions that were separated by at least 7 days to minimize carryover effects. The sessions were conducted at the same time of the day to avoid diurnal variations [25]. The tDCS was delivered for 15 min with 1 mA of current during each session [19,20,21]. During tDCS, participants performed an ankle visuomotor tracking task. The application of tDCS during motor practice was based on the results from previous studies, which have shown that the effects of tDCS are task dependent and greater when delivered in combination with a motor control task [20,26]. Corticomotor excitability (CME) was measured with TMS prior to tDCS (PRE), 10 min (POST10), and 30 min post tDCS (POST30). The accuracy of the ankle motor tracking was measured before and after tDCS.

### 2.3. Electromyography and Maximum Voluntary Contractions

For each session, the participant sat on a chair with the thigh parallel to the ground, and the knee and ankle flexed at 90°. Electromyography was collected unilaterally from the non-dominant tibialis anterior muscle with surface Ag/AgCl electrodes (Delsys Bagnoli 8, Natick, MA, USA). The participant’s skin was shaved and cleaned with alcohol to ensure adequate contact prior to the application of the surface electrode on the muscle belly. The ground/reference electrode placement was over the spinous process of the seventh cervical vertebrae. All electromyographic data were sampled at 2000 Hz, amplified *1000, and band pass filtered (10–500 Hz). The data were collected with the Spike2 software (Cambridge Electronic design, Cambridge, UK). All electromyographic activity was recorded and visualized with an oscilloscope. The maximum voluntary contractions were collected from the non-dominant tibialis anterior using a custom device with an adjustable resistance over the dorsum of the foot [20,27]. The best of three maximum voluntary contraction trials was noted. For all TMS measurements, the participants were instructed to maintain a tonic contraction that corresponded to 20% maximum voluntary contraction.

### 2.4. Transcranial Magnetic Stimulation

Single-pulse TMS was administered using a Magstim 200 stimulator (Magstim, Dyfed, Wales, UK) via a 110cm double cone coil. The coil was oriented to deliver a posteroanterior current over the lower limb M1. During TMS measurements, participants maintained ankle dorsiflexor activation at 20% MVC with visual feedback. The target tibialis anterior muscle representation (hotspot) was located as the point at which the TMS trigger elicited a maximum motor evoked potential (MEP) at the lowest stimulus intensity. For each participant, an active motor threshold was determined for the TA as the lowest stimulus intensity resulting in identifiable MEPs of at least 0.4 mV peak to peak in 50% of successive trials [27,28,29]. The CME of the tibialis anterior muscle representation was assessed using an input-output curve at seven output intensities corresponding to 80%–140% active motor threshold. Eight MEPs were recorded for each intensity at PRE, POST10, and POST30 timepoints. All TMS procedures were repeated for the second session.

### 2.5. Transcranial Direct Current Stimulation

tDCS was administered with a battery-driven constant current delivery stimulator (Chatanooga, Ionto, Hixson, TN, USA) with 1 mA of current for 15 min yielding a total charge of 0.069 C/cm^2^ [20,21]. The current density for the tDCS sessions was 0.076 mA/m^2^, which falls within the tDCS safety guidelines [30,31,32]. Prior to the application of the electrodes, the skin was scrubbed with alcohol to maximize contact.

**Figure 1:**
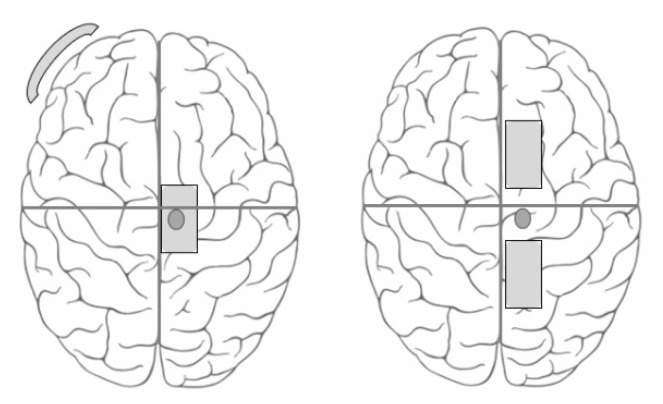
Electrode placements. Schematic representation of the two electrode configurations used. The left panel represents the Traditional electrode montage and the right panel represents the Anterior Posterior montage.

The two tDCS sessions primarily differed in the position of the electrodes (Figure 1).

(a) Traditional Electrode Montage (TRAD): The conventional electrode placement with the active electrode over the TA hotspot and the reference electrode over the contralateral supra-orbital region [19,20,21,33]. The stimulating electrode was a 13 cm^2^ (5 cm × 2.6 cm) butterfly saline-soaked electrode and the reference electrode was a 35 cm^2^ (7 cm × 5 cm) self-adhesive carbonized electrode.
(b) Anterior Posterior Montage (AP): The active electrode was placed 5 cm posterior to the tibialis anterior hotspot and the reference electrode was placed 5 cm anterior to the hotspot [18]. Both the stimulating and reference electrodes were 13 cm^2^ (5 cm × 2.6 cm) butterfly saline soaked electrodes, as it was not possible to fix the self-adhesive carbonized electrode (that was used as a reference electrode in the TRAD montage) to the scalp. The sponge electrodes were held on the scalp using a comfortably wrapped elastic bandage wrap.

### 2.6. Motor Control Task

Participants performed a skilled motor control task with their ankle while tDCS was being administered. As our target muscle for stimulation was the tibialis anterior, we chose a motor task that involved ankle dorsiflexion movement to not only better focus the tDCS currents but also to sensitively capture any changes in motor control that were elicited by tDCS. A change in the accuracy of tracking the target before and after tDCS was considered as a behavioral outcome influenced by tDCS. The ankle visuomotor task was performed with a custom-built ankle-tracking device [20,21,33]. The non-dominant leg was strapped to the ankle tracker with adjustable Velcro belts permitting free range of motion for the ankle joint in the sagittal plane (dorsiflexion and plantarflexion). Participants were instructed to track a computer-generated sinusoidal wave (random frequency between 0.2–0.4 Hz, amplitude: 60%–80% of the participant’s maximum comfortable range of motion) as accurately as possible. The motor control task during tDCS was for 15 min with a 1 min rest interval for every four minutes of movement. Two practice trials were allowed for task familiarization. Three test trials were performed before and after tDCS. The accuracy index (AI), a measure of tracking accuracy, was calculated prior to tDCS (PRE-AI) and immediately after tDCS (POST-AI).

### 2.7. Data Analyses

All data were imported and analyzed using the Spike2 software (Cambridge Electronic design, Cambridge, UK).

### 2.8. TMS

(a) MEP Changes:

The peak to peak MEP amplitude was considered as the primary measure of CME and was calculated for PRE, POST10, and POST30 time points. Raw values of the MEP amplitude were averaged across six values for each intensity for each individual. The average MEP amplitude for each time point was plotted against the corresponding intensity and the linear slope of recruitment curve was calculated. We also categorized responders and non-responders within each group. For this, we averaged the POST10 and POST30 MEP slope values to obtain a post Grand Mean (POSTGM) slope for each participant. An increase in the POSTGM slope by ≥+5% of PRE was considered as a responder and others were considered as non-responders.

(b) Ankle Motor Control:

To calculate tracking accuracy, the formula AI = 100 (P − E)/P was used, where AI is the accuracy index, E is the root-mean square (rms) error between the target line and the response line, and P is the rms value between the sine wave and the mid-line separating the upper and lower phases of the sine wave. The maximum possible score was 100%. The AI was averaged for PRE (3 trials) and POST (3 trials) time points.

### 2.9. Statistical Analyses

Statistical analyses were performed using the SPSS software (IBM version 24). A two-way repeated measures ANOVA with montage (TRAD and AP) and time (PRE, POST10 and POST30) as the within subject factors was done to examine changes in CME. A two-way repeated measures ANOVA with montage (TRAD and AP) and time points (PRE and POST) as the within subject factors was done to examine the changes in AI. *t*-tests were performed to examine the differences in PRE and POSTGM for the responders within each group and between groups for CME and AI measures.

## 3. Results

All participants completed both tDCS sessions without any adverse effects. Of the 20 participants, three outliers were excluded from the statistical analyses as they had POSTGM responses that deviated from the rest of the sample by three standard deviations in one and/or both of the tDCS sessions [34].

### 3.1. CME Changes

CME data were normally distributed as assessed by a Shapiro–Wilk’s test of normality on the studentized residuals (*p* > 0.05). Table 1 displays the percent change in CME modulation. A two-way repeated measures ANOVA revealed no significant main effects or interactions for the CME slopes of the montage (*F*_(1, 16)_ = 1.52, *p* = 0.237), time (*F*_(2, 32)_ = 0.69, *p* = 0.509), or montage × time interaction (*F*_(2, 32)_ = 0.404, *p* = 0.671). Categorization of responders and non-responders revealed ten responders and seven non-responders in TRAD, and six responders and eleven non-responders in AP. Paired sample *t*-tests between PRE and POSTGM revealed significant upregulation for the TRAD (t = 2.57, *p* = 0.03) and AP (t = 3.18, *p* = 0.024) groups. An independent *t*-test to compare the POSTGM slopes between responders of both groups revealed no significant differences (t = 1.36, *p* > 0.05). A non-significant trend towards greater facilitation in the TRAD group was noticed for the entire sample as well as the responders.

### 3.2. Ankle Motor Control

Table 2 displays the data for the PRE and POST AI of the two groups. The two-way repeated measures ANOVA revealed no significant main effects or interactions for changes in the AI of the montage (*F*_(1, 16)_ = 0.37, *p* = 0.54), time (*F*_(2, 32)_ = 3.4, *p* = 0.06), or montage × time (*F*_(2, 32)_ = 0.01, *p* = 0.91). Paired *t*-tests for AI changes within the TRAD group revealed significant improvement for responders (t = −2.96, *p* = 0.016) but not non-responders (t = −0.02, *p >* 0.05). For the AP group, there was no significant improvements for the responders (t = −2.05, *p >* 0.05) or non-responders (t = −4.74, *p >* 0.05). Independent *t*-tests to compare AI changes in responders between the TRAD and AP montage groups revealed no significant differences (t = 0.56, *p >* 0.05).

## 4. Discussion

This study aimed to determine if the anterior–posterior placement of the tDCS electrodes elicits superior effects compared to the conventional montage for the lower limb motor cortex in healthy individuals. Contrary to our hypothesis and what has been postulated empirically [18], the AP montage did not reveal a higher CME or motor control change compared to the TRAD montage. Similar to previous studies in healthy adults [19,20,35], there were no statistically significant differences in the CME for both groups when all subjects were considered. Changes in CME were significant when individuals were categorized into responders and non-responders. The number of responders in the TRAD group was higher than the AP group (10 vs. 6). Although we observed the magnitude of the CME to be greater for the responders in the TRAD group, there were no statistical differences between the two groups. Changes in motor control also followed the trends for CME; no group effect was observed. However, only responders in the TRAD group demonstrated significant improvements in motor control from baseline.

There has been a recent surge of studies addressing the critical need to optimize the neuromodulatory effects of tDCS and reduce the inter-individual variability that is commonly observed with neurophysiological measures. Numerous studies have suggested that the conventional tDCS montage may induce suboptimal neuromodulation, and researchers have proposed alternate montages based on empirical data [11,17,18,36]. Finite element modeling provides useful information regarding current flow, but experimental validation of neurophysiological and behavioral effects is necessary to proceed with clinical implementation. Projected mathematical findings do not account for individual biological parameters such as the orientation of neurons, attention, or fatigue that may affect tDCS currents in vivo. In this study, we attempted to validate a new anterior–posterior electrode placement, which has been proposed to be more powerful than the traditional montage [17,22,37]. The results of our study do not confirm the superiority of this new montage. Although some individuals showed upregulation with the AP montage, the frequency and magnitude were lesser than the conventional montage.

When comparing the two electrode montages, it is important to consider some of the defining physical characteristics that shape the flow of current. Current density, the amount of electric current per cross sectional area, is an important parameter to determine optimal neuromodulation [27]. We used an anodal stimulating electrode size of 13 cm^2^ for both the TRAD and AP placements. We intentionally chose a smaller electrode area for the lower limb M1 to provide greater focality of tDCS [10,19,21,22,33]. It is important to note that the size of the reference electrode was different between the two groups. For the TRAD group, a larger conventional reference electrode (35 cm^2^) was used. A larger cathode has been proposed to facilitate asymmetrical current density distribution, with a more effective distribution under the smaller electrode [38,39]. We were unable to use the larger reference for the AP montage as the material of the electrode prevented adherence to the scalp. Hence, the electrode used for the anode was used for the reference as well. This may partly explain the relatively higher neuromodulation for the TRAD group. The inter-electrode distance is another parameter that affects the flow of currents. A greater inter-electrode distance is suggested to reduce the shunting of the current through the scalp and increase the current density under the edges of the smaller electrode [38]. The inter-electrode distance for the AP group (10 cm) was lesser than that of the TRAD group (~20 cm). It is also important to acknowledge that as the distance between the electrodes increases, the current intensity must also be adjusted to achieve the same resultant current density under the stimulating electrode [39,40]. In this study, we maintained the same current intensity between the two groups, perhaps resulting in a more conservative modulation for the TRAD group considering the interelectrode distance was greater.

Similar to some previous studies, we did not find the main effect of tDCS on neurophysiological changes when all participants were considered together [6]. The lack of significance could be attributed to the high inter-individual variability that is typically seen with tDCS. tDCS may modulate neuronal circuits differently for individuals depending on biological factors, such as the orientation of neurons, genotype, circadian rhythm, hormones, menstrual cycle, hair, and skull thickness [25,41,42,43]. Some individuals have a significantly higher or lower potential for neuromodulation in relation to others in a group, and these values are often masked by group data averaging [7,8,9,10]. Hence, we characterized our CME data into responders and non-responders to obtain a better understanding of tDCS effects. Our rate of responders in this study was similar to the 40%–60% response reported in previous studies. Ten out of 17 participants showed significant facilitation of CME in the TRAD group, and six out of 17 participants showed facilitation in the AP group. When the data were categorized according to responsiveness, we found that responders showed significantly higher modulation than baseline for both groups. The minimal facilitatory effects of tDCS and the clear lack of superiority of one montage over the other may also be explained by the inability of an already optimally excited healthy motor system to modulate further [44]. The effects of montages may have to be tested in a neurologically impaired system to truly determine the optimal stimulation parameters for tDCS.

Given the variant nature of neurophysiologic measures, we included a behavioral task to evaluate the effects of the two montages. Changes in tracking accuracy reflected the insignificant changes seen in CME in the whole group. When participants were dichotomized based on responsiveness to tDCS, changes in motor control were greater and statistically different from baseline only for the responders in the TRAD group. This effect may reflect the trend for greater CME facilitation seen in the responders of the TRAD group.

The mechanisms of action for tDCS are still under consideration. Although the physiological effects of tDCS have been suggested to be a result of change in neuronal membrane potential resulting in changes in synaptic plasticity, alternative mechanisms (such as the possible contribution of the sensory nerves) cannot be ruled out. It has been suggested that a large part of the current goes through the scalp from the anode to the cathode without reaching the deeper neurons [18]. Thus, it is possible that sensory scalp stimulation is responsible for some of the changes in neuromodulation seen in our study. It is also possible that the maximal area of stimulation is located elsewhere in the brain, such as the frontal areas (for the conventional montage) and the supplementary motor area (for the anterior–posterior montage) [18,45,46]. Another hypothetical explanation is that any small change in the homeostatic balance of neuronal excitability (as elicited with tDCS) may be sufficient to cascade a change in neural networks and thereby improve motor behavior.

The results of this study must be interpreted with the following caveats. Our choice of reference electrode size was different between the two groups. We intentionally chose a larger reference electrode for the traditional montage to keep in line with what has been used conventionally in lower limb tDCS studies. The current density and patterns of distribution of the currents are dependent on the shape and size of electrodes and may have created dissimilar electric fields that limit comparisons between the two groups. Future studies using similar electrode sizes are preferable. We validated the two montages in healthy brains, and this may not transfer to persons with brain lesions who may show a different shunting of current flow than healthy participants. The interpretations of the results are also limited by the small sample size. Further studies warrant larger sample sizes especially when identifying responders to tDCS and multiple sessions to understand the cumulative effects. Furthermore, we did not include a sham condition (only motor task) or provide tDCS at rest, which may limit the interpretation of changes in the corticomotor excitability truly induced by tDCS and not elicited by motor activity. Although a single study team member placed the electrodes for all participants to ensure consistency, results may have been compromised by slight inaccuracies in manual measurements. Navigated electrode placements in addition to personalized brain network models might be helpful to reduce such variability in the future [12].

## 5. Conclusions

This is the first study to compare the neurophysiological and behavioral changes between the traditional and anterior–posterior montages. The results of this study do not validate the superiority of the anterior–posterior montage for tDCS. Further work is needed to determine the best electrode montages to optimize the effects of tDCS.

## Figures and Tables

**Table 1:** Corticomotor excitability.

	Traditional		Anterior–Posterior	
	*N*	Change in CME	*N*	Change in CME
All	17	9.4 ± 7.0%	17	−0.6 ± 3.0%
Responders	10	36 ± 10.0% *	6	18 ± 3.2% *
Non-responders	7	−12 ± 4.3%	11	−11 ± 2.0%

Comparison of changes in corticomotor excitability (CME) between the two electrode placements. POST GM slopes for responders within each group was significantly different from PRE. * *p* < 0.05. CME, corticomotor excitability.

**Table 2:** Ankle motor control.

	Traditional		Anterior–Posterior	
	*N*	Change in AI	*N*	Change in AI
All	17	6.4 ± 2.85%	17	7.19 ± 1.37%
Responders	10	14.6 ± 4.9% *	6	8 ± 3.9%
Non-responders	7	0.09 ± 3.4%	11	6.6 ± 1.3%

Comparison of tracking accuracy, measured as the accuracy index (AI), between the two groups. Responders showed significantly increased change from PRE for the TRAD group. * *p* < 0.05.
